# Systematic Review and Meta-Analysis of Good Self-Care Practice among People Living with Type 2 Diabetes Mellitus in Ethiopia: A National Call to Bolster Lifestyle Changes

**DOI:** 10.1155/2021/8896896

**Published:** 2021-02-20

**Authors:** Baye Dagnew, Getu Debalkie Demissie, Dessie Abebaw Angaw

**Affiliations:** ^1^Department of Human Physiology, School of Medicine, College of Medicine and Health Sciences, University of Gondar, P. O. Box 196, Gondar, Ethiopia; ^2^Department of Health Promotion and Behavioral Sciences, Institute of Public Health, College of Medicine and Health Sciences, University of Gondar, P. O. Box 196, Gondar, Ethiopia; ^3^Department of Epidemiology and Biostatistics, Institute of Public Health, College of Medicine and Health Sciences, University of Gondar, P. O. Box 196, Gondar, Ethiopia

## Abstract

**Background:**

Self-care practice is the mainstay of management for good glycemic control. Despite the presence of a few pocket studies, no comprehensive study was conducted in Ethiopia to demonstrate the overall good self-care practice among diabetic patients in Ethiopia. Therefore, we intended to conduct this systematic review and meta-analysis to estimate the overall good self-care practice among people living with type 2 diabetes mellitus (T2DM) in Ethiopia.

**Methods:**

We systematically searched PubMed, Scopus, Science Direct, Cochrane library, Google scholar, and direct Google to retrieve relevant studies. Forest plot was used to present the pooled estimate of good self-care practice using DerSimonian and Laird's random-effects model. We checked publication bias using Egger's test and funnel plot. Potential heterogeneity was tested using the I-squared statistic. Moreover, subgroup and sensitivity analyses were performed.

**Results:**

In this review, 12 primary studies (with a total sample size of 4030) were included. Because of the presence of heterogeneity, we employed a random-effects model. After running the random-effects model, the pooled estimate of overall good self-care practice was 51.12% (95% CI: 41.90–60.34). Furthermore, the pooled estimate of good dietary practice was 50.18% (95% CI: 32.75–67.60), good physical exercise practice was 48.29% (95% CI: 34.14–62.43), the good footcare practice was 63.61% (95% CI: 45.56–81.66), and appropriate self-monitoring of the blood glucose level was 31.89% (95% CI: −4.62–68.41). In this meta-analysis, there was serious interstudy variation, but there was no publication bias.

**Conclusions:**

The overall good self-care practice among people living with T2DM was low which necessitates the need for designing strategies to increase the self-care practice. The health sector has to bolster awareness creation to allow better plasma glucose control and preventing diabetes-related complications. This trial is registered with CRD42019147694.

## 1. Background

Diabetes mellitus (DM) is a silently progressive, but serious health condition which affects 386.7 million (prevalence of 8.3%) people globally and projected to rise to 592 million by 2035 [1]. In developed countries, the prevalence of diabetes mellitus is closer to 16% [2]. As reported in 2012, above 77% of diabetes-related morbidity and 88% of mortality occurred in low- and middle-income countries [2, 3]. This is profoundly increased in developing countries, which is attributed to the economic growth, increased life expectancy, and lifestyle changes associated with increasing trends towards urbanization, including unhealthy diet, obesity, and a sedentary lifestyle, resulting in late-onset DM [4]. In Ethiopia, the prevalence of DM was 3.5% in 2011 [5]. The extrapolated prevalence in 2013 was 4.36%, and 34,262 DM patients died in Ethiopia in 2013 [1] with the projected number of DM cases to upsurge to 1.8 million in 2030 [6].

Diabetes-related complications are the major causes of morbidity and mortality [5] and reduce the patients' quality of life and productivity [7]. Almost no organ is free of diabetes-related complications, but mainly, the eyes (retinopathy, blindness), kidneys (nephropathy), and nerves (neuropathy), blood vessels, musculoskeletal system (poor wound healing), and oral cavity (periodontal diseases) are affected [8].

These vast bad outcomes of DM can only be averted by the cooperative actions of patient activities and health workers intervention [9]. Among the patients' side, a self-care practice is the mainstay of management for the prevention of diabetes-associated complications and improves health outcomes of patients. Individual components of self-care practice include healthy eating, blood glucose monitoring, physical exercise, foot-care, and healthy coping [10]. Good self-care practice in DM is associated with improved glycemic control [11–13]. Physical exercise for at least 30 minutes a day can reduce diabetes-associated cardiovascular risks by 50% [14].

Even though pieces of evidence showed that good self-care practice is essential to improve the clinical outcomes of DM [15], there is no comprehensive study in Ethiopia to determine self-care practice at the national level. Therefore, this systematic review and meta-analysis aimed to determine the pooled estimate of overall good self-care practice among people living with T2DM in Ethiopia.

## 2. Methods

### 2.1. Reporting and Registration of the Protocol

The reporting approach was using the Preferred Reporting Items for Systematic Review and Meta-Analysis guideline [16]. We registered the protocol at PROSPERO with protocol number CRD42019147694 and is available at https://www.crd.york.ac.uk/PROSPERO.

### 2.2. Inclusion Criteria

- Setting: studies conducted in Ethiopia and its localities
- Population: adult people living with T2DM
- Study type: studies that applied cross-sectional design that reported proportion or prevalence of good self-care practice
- Outcome: good self-care practice among adult people living with T2DM
- Publication status: all published studies
- Publication year: no restriction of publication year
- Publication date: we included studies published until 5 May 2019
- Language: all studies published in English only
- Exclusion criteria: citations without abstract, case-reports, case series, unidentified reports, editorials, and those qualitative studies, studies that reported sex-specific findings, either male-only or female-only, were excluded.

### 2.3. Data Sources

We systematically searched electronic databases including PubMed, Scopus, Science Direct, Cochrane library, and also Google Scholar, and direct Google search to find out primary studies that are relevant to the systematic review and meta-analysis of good self-care practice among people living with T2DM.

### 2.4. Searching Strategies

In our searching strategy, we included a combination of words or phrases interrelated to self-care practice among adults living with T2DM. The basic search terms and phrases were “self-care,” “self-adherence,” “behavior,” “practice,” “adherence,” “dietary behavior,” “self-management,” “self-control,” “diabetes,” “type 2 diabetes mellitus,” “diabetes mellitus,” and “hyperglycemia.” Corresponding authors were contacted to get full-texts for those studies with incomplete data. We also retrieved additional relevant studies by searching the reference lists of included studies to account for missed studies during electronic database searching. To fit advanced search for different electronic databases, we used a combination of MeSH (Medical Subject Heading) terms or phrases using Boolean operators, “AND” and “OR.” For PubMed database searching, we used the following search strategy: (self-care [Title/Abstract]) OR practice [Title/Abstract]) OR behavior [Title/Abstract]) OR adherence [Title/Abstract]) AND “type 2 Diabetes mellitus ”[Title/Abstract]. For SCOPUS database searching we applied a combination of [TITLE-ABS-KEY] (self-care) AND [TITLE-ABS KEY] (behavior OR practice OR adherence) AND [TITLE-ABS-KEY] (type 2 diabetes AND Mellitus OR diabetes OR insulin AND independent AND diabetes).

### 2.5. Selection of Studies

We exported all the retrieved primary studies into Endnote version 7 reference manager to remove duplicate studies and also to manage in-text citation and bibliography. Two independent reviewers (BD and GDD) performed screening of the included studies using specific selection criteria. Most of the discrepancies were resolved by the two (BD and GDD) reviewers by consensus after thorough discussion. Few disagreements between the two reviewers were harmonized by the third reviewer (DAA) based on conventional article selection criteria.

### 2.6. Risk of Bias Assessment

All the selected studies for inclusion were appraised independently by the two authors (BD and GDD) using the quality assessment tool adapted from Hoy et al. [17] that can address both the external and internal validities. The tool has 9 risks of bias items with a maximum score of nine and minimum score zero. The overall risk of study bias has 3 parts such as low risk (0–3) [2], moderate risk [4–6], and high risk [7–9].

### 2.7. Data Abstraction

Two authors (BD and GDD) extracted the data independently using a structured data extraction format prepared by Microsoft 2010 Excel spreadsheet. After independent extraction, repetition was performed for any variations in the extraction process. Most discrepancies were fixed by the two authors (BD and GDD), and few discrepancies were resolved by the third reviewer (DAA). We communicated with corresponding authors of eligible studies through e-mail and/or cellphone to access incomplete data. The following data were extracted for each included studies: the name of the corresponding author, publication year, study design, study locality, sample size, study type, overall good self-care practice, and individual good self-care practices.

### 2.8. Outcome Measurement

The primary outcome was good self-care practice among people living with T2DM. Besides, good dietary practice, good footcare practice, self-monitoring of blood glucose, and appropriate physical exercise were the outcome variables for this review. Self-care practice is measured using the Summary of Diabetes Self-Care Activities (SDSCA) which contains four domains such as dietary, footcare, exercise, and self-monitoring of blood glucose in the last 7 days. Response choices range from 0 to 7. The overall mean score was calculated by summation of each item of the scales and divided by the number of items. Therefore, participants who scored equal to or greater than a mean score were classified as having good diabetes self-care practice. Self-care practice is self-management undertaken by the patients' willingness and consisted of four domains such as dietary, footcare, exercise, and self-monitoring of blood glucose [18, 19].

Good dietary practice: using the modified form of the fourteen item scales, patients who scored above the median value for the response were classified as having good dietary practice [20].

Good physical exercise practice: 20–30 min of aerobic exercise such as walking or swimming 3-4 days per week [19].

Good footcare practice: patients who scored a total practice score of ≥50% of the maximum score are considered as having good footcare practice.

Appropriate self-monitoring of the blood glucose level: A diabetic patient who monitored his blood glucose level at least four times a day is considered as having good self-monitoring of the blood glucose level [21].

### 2.9. Reliability

A second reviewer (GDD) was blinded to primary reviewer's (BD) decision for checking articles selection, data extraction, and risk of bias assessment phases of the review. Any dissimilarities of judgment were discussed; otherwise, a third reviewer (DAA) was available to arbitrate any issues that remained unresolved.

### 2.10. Statistical Analysis

The analysis was performed by the active participation of three authors (BD, GDD, and DAA). We performed Egger's test and funnel plot to find out the possibility of publication bias. I-squared statistics were computed to check heterogeneity in which 25%, 50%, and 75% represent low, moderate, and high heterogeneity, respectively [22]. The pooled self-care practice was estimated using DerSimonian and Laird's random-effects model [23]. Subgroup analysis was performed to resolve study variations. Sensitivity analysis was employed to identify possible outlying studies and the effects of a single study on the overall estimation. Stata version 11 was used for the analysis with installed meta-analysis packages. The measure of the outcome variable was point estimate with 95% interval estimate.

## 3. Results

### 3.1. Characteristics of Included Studies

**Figure 1:**
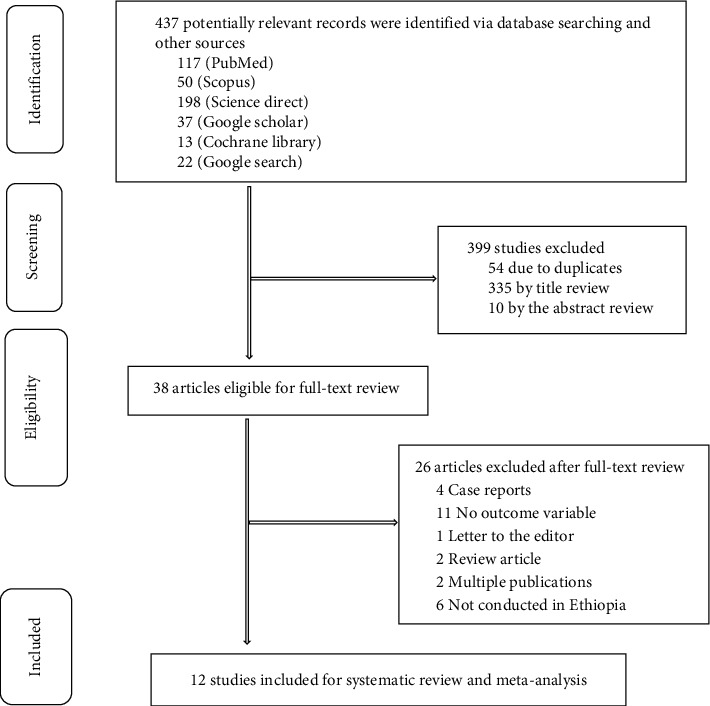
A PRISMA flow diagram exemplifying the study selection process of included studies for systematic review and meta-analysis of good self-care practice among people living with T2DM.

After full searching of major electronic databases, we retrieved 437 studies, of which 12 studies [24–35] (with a total sample size of 4030 adults living with T2DM) were included for the qualitative synthesis and meta-analysis of good self-care practice (Figure 1).

Of the included studies, the sample size ranged from 194 [35] to 637 [33]. We planned to include all observational studies (case-control, cohort, and cross-sectional studies), but we found only cross-sectional studies which had the required outcome variable and all of them were published. The quality assessment was performed using Hoy et al. critical appraisal tool in each study with no considerable risk of bias for each study. All studies used adults aged 18 years and above except one study which used those participants with the age above 15 years. Regarding sex composition, only 11 studies described the sex compassion as male and females, but one study did not show the male to female ratio (Table 1).

The pooled estimate of good self-care practice among people living with T2DM.

**Figure 2:**
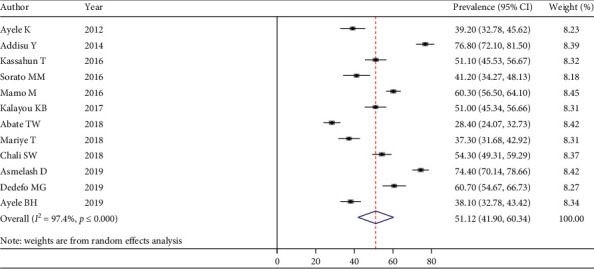
Estimated pooled prevalence of overall good self-care practice among T2DM.

In the meta-analysis of the magnitude of overall good self-care practice, there was substantial heterogeneity (I^2^ = 97.4%). To account for this interstudy variation, we used a random-effects model for the main meta-analysis of estimated pooled prevalence of overall good self-care practice, and it was 51.12% (95% CI: 41.90–60.34) (Figure 2).

### 3.2. Subgroup Analysis

We performed subgroup analysis by year of publication with still high heterogeneity. The estimated pooled prevalence of good self-care practice was highest in studies published before 2017 (53.88%), and it was 54.37% in those studies who used a sample size of above 384 (Table 2).

### 3.3. Publication Bias

**Figure 3:**
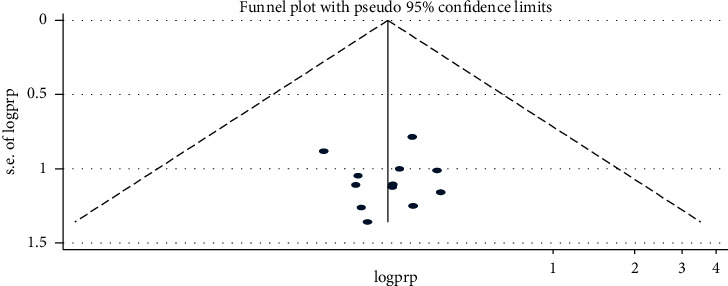
Funnel plot to show publication bias for the good self-care practice.

It was assessed using funnel plot, and the result indicated the symmetrical distribution of studies (Figure 3) and Egger's regression test (*p* > 0.05) with bias coefficient = 0.018 indicating that there was no publication bias.

### 3.4. Sensitivity Analysis

**Figure 4:**
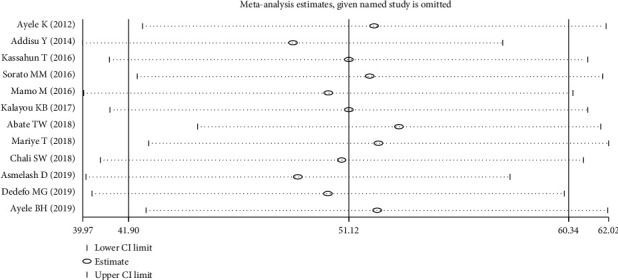
Sensitivity analysis for studies included in the meta-analysis of overall good self-practice.

To check the effect of a single study on the overall outcome, we used the leave-one-out method, and there was no observed study which exerted a significant impact on the overall estimate of good self-care practice (Figure 4).

### 3.5. The Estimated Pooled Prevalence of Individual Good Self-Care Practice

**Figure 5:**
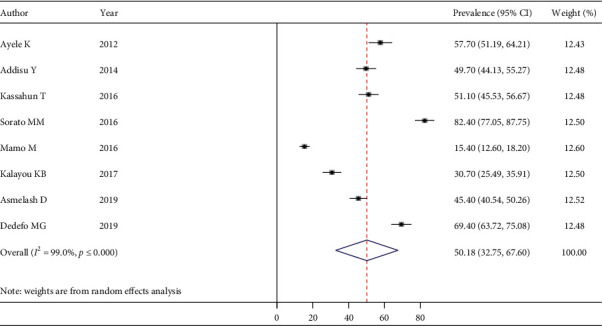
Estimated pooled prevalence of good dietary practice among T2DM.

**Figure 6:**
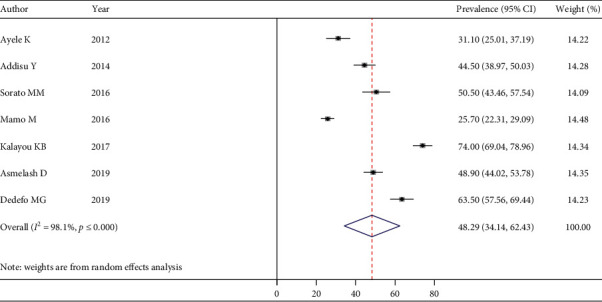
Forest plot depicting good physical exercise among T2DM.

**Figure 7:**
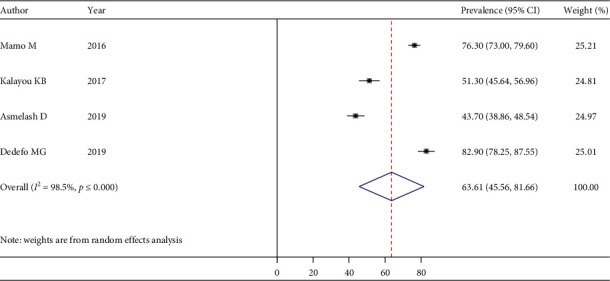
Pooled estimate of good footcare practice among people living with T2DM.

**Figure 8:**
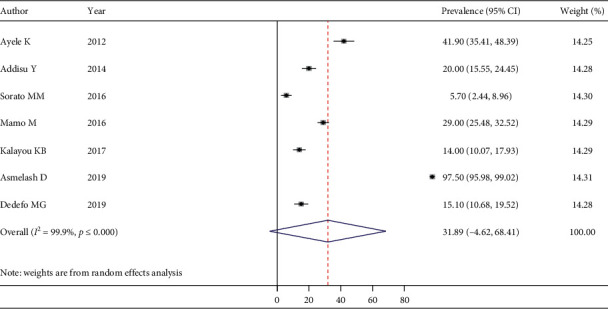
Pooled estimate of appropriate self-monitoring of the blood glucose level among T2DM.

The pooled estimate of good dietary practice was 50.18% (95% CI: 32.75–67.60) (Figure 5), good physical exercise practice was 48.29% (95% CI: 34.14–62.43) (Figure 6), good footcare practice was 63.61% (95% CI: 45.56–81.66) (Figure 7), and appropriate self-monitoring of the blood glucose level was 31.89% (95% CI: -4.62–68.41) (Figure 8).

## 4. Discussion

Due to lack of shreds of evidence on the overall good self-care practice, this systematic review and meta-analysis aimed to determine the estimated pooled prevalence of overall good self-care practice and its components (dietary practice, physical exercise, blood glucose monitoring, and footcare practices) among people living with T2DM. Self-care practice is a vital strategy for the management of diabetes mellitus using different domains (components) of self-care practice such as good dietary practice, physical exercise, footcare, and self-monitoring of blood glucose level [36]. Self-care practice is important for healthcare sector stakeholders to design and implement prevention strategies for DM-related complications [37]. The practice of self-care is not only important to diabetic patients but also for other medical practices to reduce the direct and indirect costs of medicine [38]. Plasma glucose control is important for prevention and decelerating the progression of complications which can be achieved by integrated management (pharmacologic and nonpharmacologic) [39].

In this meta-analysis, the pooled estimate of overall good self-care practice among people living with T2DM in Ethiopia was 51.12% (95% CI: 41.90–60.34). This is similar to a study conducted in Malaysia (51.12 vs. 53%) [40] but lower than a study in Bangladesh (51.12% vs. 71.8%) [41]. This difference might be due to the socioeconomic status of the population, extent (scope) of the study, and sample size variations. Furthermore, the status of health service delivery in the country could affect self-care practice. Healthcare providers and the community education level, media exposure level, and utilization of standard guidelines to practice self-care are also factors which might bring differences in the level of self-care practice among different countries. The pooled estimate of good physical exercise in this meta-analysis was lower than a study in Ghana (48.29% vs. 69.33%) [42] but higher than a study in Yemen (48.29% vs. 15.2%) [43] and Poland (48.29% vs. 26.5%) [44]. This difference might be because of sample size differences and wealth index of the countries. Besides the abovementioned reasons, the health system of the country and community education might affect the practice. Regarding good dietary practice, the pooled estimate was 50.18% which is consistent to a study in Ethiopian hospitals (50.18% vs. 44.3%) [45], Addis Ababa, Ethiopia (50.18% vs. 48.6%) [46], and Bahir Dar, Ethiopia (50.18% vs. 35.9%) [20] but higher than a study conducted in Yemen (50.18% vs. 21.0%) [43]. This difference might be justified by variations in the population of the study, duration of diabetes mellitus, and health information given by health educators or health service providers. The pooled estimate of good footcare was 63.61% which is higher than a study in Malaysia (63.61 vs. 38.2%) [47], Iraq (63.61% vs. 40%) [48], and Gujarat, India (63.61% vs. 16.6%) [49]. This could be due to the variations in the socioeconomic status, the extent of the study, and sample size differences. Moreover, the composition of healthcare providers could affect footcare practice. As it is known, the prevalence of T2DM and its complications are progressively rising. This study is implicated to the Ethiopian Federal Ministry of Health and local health officials to monitor and undertake supportive supervision to health professionals and service delivery to prevent long-term complications of T2DM.

### 4.1. Limitations of the Study

As a limitation, the results of this study should be interpreted by considering possible limitations. Due to lack of sufficient studies in Ethiopia, it might be difficult to generalize to the whole population. Interstudy heterogeneity was profound in this review which was not resolved using subgroup analysis, and we were not able to find out the remarkably influencing factors for the outcome.

## 5. Conclusions

This systematic review and meta-analysis revealed a lower pooled estimate of overall good self-care practice of which good footcare practice was the highest. Self-care practice is profoundly important for the control of plasma glucose concentration among people living with T2DM. The health sector stakeholders have to bolster awareness creation to enhance self-care practice of people living with T2DM to bring better plasma glucose control and preventing diabetes mellitus associated complications.

## Figures and Tables

**Table 1:** Characteristics and quality status of the included studies for this systematic review and meta-analysis.

Author	Year	Country	Study design	Sample size	Sex	Age (years)	Good dietary practice (%)	Good physical exercise	Good footcare practice (%)	Good self-monitoring of blood glucose level (%)	Overall good self-care practice (%)	Quality status
Kassahun T [32]	2016	Ethiopia	Cross-sectional	309	*M* = 189	≥18	51.1	—	—	—	51.1	Low risk
Chali SW [29]	2018	Ethiopia	Cross-sectional	383	*M* = 209	≥18	—	—	—	—	54.3	Low risk
Asmelash D [25]	2019	Ethiopia	Cross-sectional	403	*M* = 216	≥18	45.4	48.9	43.7	97.5	74.4	Low risk
Abate TW [24]	2018	Ethiopia	Cross-sectional	416	*M* = 240	≥18	—	—	—	—	28.4	Low risk
Mariye T [34]	2018	Ethiopia	Cross-sectional	284	NA	≥18	—	—	—	—	37.3	Low risk
Sorato MM [35]	2016	Ethiopia	Cross-sectional	194	*M* = 95	>15	82.4	50.5	—	5.7	41.2	Low risk
Kalayou KB [28]	2017	Ethiopia	Cross-sectional	300	*M* = 173	≥18	30.7	74.0	51.3	14.0	51	Low risk
Mamo M [33]	2016	Ethiopia	Cross-sectional	637	*M* = 290	≥18	15.4	25.7	76.3	29.0	60.3	Low risk
Ayele K [27]	2012	Ethiopia	Cross-sectional	222	*M* = 88	≥18	57.7	31.1	—	41.9	39.2	Low risk
Ayele BH [26]	2019	Ethiopia	Cross-sectional	320	*M* = 142	≥18	—	—	—	—	38.1	Low risk
Addisu Y [31]	2013	Ethiopia	Cross-sectional	310	*M* = 200	≥18	49.7	44.5	—	20.0	76.8	Low risk
Dedefo MG [30]	2019	Ethiopia	Cross-sectional	252	*M* = 138	≥18	69.4	63.5	82.9	15.1	60.7	Low risk
The total sample size of the 12 studies				4030	*M* = 1980							

*M,* male; NA, not available (from the included studies, one study did not describe the number of males and females in the study).

**Table 2:** Subgroup analysis of the pooled estimate of good self-care practice with 95% CI, *p* value, and heterogeneity estimate among people living with T2DM.

Subgroup analysis by	Characteristics	Pooled estimate (%)	*I* ^2^ (*p* value)
Year of publication	Before 2017	53.88 (40.77–66.98)	96.8% (*p* ≤ 0.001)
	≥2017	49.17 (36.16–62.18)	97.8% (*p* ≤ 0.001)

Sample size	≤384	50.03 (40.85–59.21)	95.9% (*p* ≤ 0.001)
	>384	54.37 (28.73–80.05)	99.1% (*p* ≤ 0.001)

## Data Availability

The dataset used to support the findings of this study is available from the corresponding author upon request.

## Abbreviations

CI: Confidence interval
DM: Diabetes mellitus
ES: Effect size.
